# Recommendations for the measurement of sexual steroids. A step forward in the silent revolution of mass spectrometry

**DOI:** 10.1515/almed-2023-0022

**Published:** 2023-03-10

**Authors:** Joan Lopez Hellin

**Affiliations:** Servicio de Bioquímica Clínica, Hospital Universitari Vall d’Hebron, Vall d’Hebron Barcelona Hospital Campus, Barcelona, Spain

Mass spectrometry has been demonstrated to be superior to traditional immunoassays when sexual steroids have to be measured in specific conditions. These contexts include the measurement of steroids at low concentrations or in the presence of interfering factors (recommendations published in this Issue [1]). Yet, this is not an isolated situation, as when traditional immunoassays are ineffective, mass spectrometry emerges as the solution. A clear example is the measurement of levels of immunosuppressant drugs in blood, where mass spectrometry is gaining ground over immunoassays. Why is this happening?

When we talk about the use of mass spectrometry for the measurement of steroids, we refer to liquid chromatography tandem-mass spectrometry (LC-MS/MS) with isotopic dilution. LC-MS/MS is considered one of the best quantification techniques currently available. When operated by experienced hands, this technique is not only the gold standard but, according to the IUPAC, LC-MS/MS is a definitive method, the ultimate measurement technique, with no room left for improvement. The reason is that LC-MS/MS is not an indirect quantification method based on a derived property, such as light emitted by an antibody binding or not a substance. Instead, LC-MS/MS directly measures a basic physical property of molecules: mass. LC-MS/MS, together with isotopic dilution, has an almost absolute specificity and a very good sensitivity, which continues to improve as technology evolves. As a result, its capacity of measurement is unbeatable. Last but not least, LC-MS/MS is an almost universal method, as it virtually quantifies any molecule, ranging from glucose and proteins to drugs. This universal technology makes it possible to measure several analytes simultaneously without it involving a higher cost. As a result, large panels of hormones, drugs, and biomarkers can be quantified in a single sample [2].

Here, the question arises, why is LC-MS/MS not implemented in all laboratories? Unfortunately, there are some factors that limit the widespread use of this technique. Firstly, LC-MS/MS equipment is technically very complex and requires manual operation. Every change of method requires “re-engineering” the equipment. Thus, the use of LC-MS/MS instruments requires the availability of specialized technical personnel for sample processing, process monitoring, and maintenance. Additionally, highly-qualified personnel are also needed to fine-tune the equipment and methods, detect and correct problems and analyze results. Secondly, although reagents are not expensive, the equipment is. The acquisition of the equipment and annual maintenance involve a high cost. Finally, although the speed of sample processing is high, bulk speed decreases when big sample batches are quantified, as samples are processed sequentially, not in parallel as in immunoassays. Therefore, LC-MS/MS cannot compete with automated analyzers in terms of speed [3]. These pitfalls explain reluctance of clinical laboratories to incorporate mass spectrometry, especially when highly competent staff with previous experience in this field is not available. Thus, the availability of robustly trained staff is crucial for LC-MS/MS technology to be successfully implemented in the clinical laboratory.

Fortunately, technical advances and positioning of the industry are turning the tide, as the advantages of this technique increase and its drawbacks are overcome. As a result, an increasing number of laboratories are installing LC-MS/MS analyzers [4]. LC-MS/MS instruments are increasingly sensitive, rapid and easy-to-use. Manufacturers of mass spectrometry instruments, traditionally involved in the research and industry fields, have now a better understanding of clinical needs. Consequently, they are betting on the use of simpler automated instruments. They are making efforts to adapt to this segment and provide maintenance services that meet the needs of the clinical laboratory. In addition, manufacturers are adapting their customer support services to users without previous significant experience with mass spectrometry. These changes will progressively facilitate the widespread use of mass spectrometry in the clinical laboratory, although some significant pitfalls remain unsolved. Nevertheless, although a considerable effort is required, the reward is huge. Mass-spectrometry improves the quality and reliability of results significantly. Based on our experience with immunosuppressant drugs, the implementation of mass spectrometry has been enthusiastically received by physicians, who would hardly agree to return to immunoassays.

If the present is encouraging, the near future is looking even brighter, since the industry of diagnostics is considering including mass-spectrometry in their analyzers. This may represent a revolution in the clinical laboratory: fully-automated, integrated LC-MS/MS analyzers adapted to the automated core laboratory that operate as conventional analyzers. In this context, highly-trained personnel or complex fine-tuning, analysis or manual maintenance processes would not be required. LC-MS/MS technology has come to complement immunoassays when the sensitivity or specificity of the latter is insufficient, at the price of a lower throughput speed and higher cost. This technology will be especially useful when measuring levels of immunosuppressant drugs, estradiol and testosterone at low concentrations, or in the presence of interfering factors. Other uses include the quantification vitamin D in special cases, confirmation of the presence of abuse drugs in blood, or the measurement of antimycotic, antibiotic, and antiepileptic drugs, which are currently determined by liquid chromatography.

The recommendation of using LC-MS/MS for the determination of sexual steroids in special contexts is a step forwards on the long no-return path towards the adoption of mass spectrometry in the clinical laboratory. In the future, automated mass-spectrometry instruments integrated in core laboratories will coexist with conventional LC-MS/MS equipment, reserved for laboratories of reference. In these laboratories, conventional LC-MS/MS will be used when automated analyzers are not available for measuring a specific molecule. We are living a similar experience as when RIA, western blot and ELISA were the only immunoassays available and immunochemistry analyzers emerged as a new solution. At that moment, we could hardly imagine that core laboratories would have integrated biochemistry and immunochemistry analyzers operating at high speed. In the coming years, core laboratories will have integrated biochemistry, immunochemistry and mass spectrometry analyzers, each with their specific functions, which will revolutionize the analytical capacity of clinical laboratories.
